# Restoring Specific Lactobacilli Levels Decreases Inflammation and Muscle Atrophy Markers in an Acute Leukemia Mouse Model

**DOI:** 10.1371/journal.pone.0037971

**Published:** 2012-06-27

**Authors:** Laure B. Bindels, Raphaël Beck, Olivier Schakman, Jennifer C. Martin, Fabienne De Backer, Florence M. Sohet, Evelyne M. Dewulf, Barbara D. Pachikian, Audrey M. Neyrinck, Jean-Paul Thissen, Julien Verrax, Pedro Buc Calderon, Bruno Pot, Corinne Grangette, Patrice D. Cani, Karen P. Scott, Nathalie M. Delzenne

**Affiliations:** 1 Metabolism and Nutrition Research Group, Louvain Drug Research Institute, Université catholique de Louvain, Brussels, Belgium; 2 Toxicology and Cancer Biology Research Group, Louvain Drug Research Institute, Université catholique de Louvain, Brussels, Belgium; 3 Laboratory of Cell Physiology, Institute Of NeuroScience, Université catholique de Louvain, Brussels, Belgium; 4 Gut Health Division, Rowett Institute of Nutrition and Health, University of Aberdeen, Aberdeen, United Kingdom; 5 Departement of Diabetology and Nutrition, Institut de recherche expérimentale et clinique, Université catholique de Louvain, Brussels, Belgium; 6 Lactic Acid Bacteria and Mucosal Immunity, Center for Infection and Immunity of Lille, INSERM U1019-CNRS UMR 8204, Institut Pasteur de Lille, Université Lille Nord de France, Lille, France; Charité, Campus Benjamin Franklin, Germany

## Abstract

The gut microbiota has recently been proposed as a novel component in the regulation of host homeostasis and immunity. We have assessed for the first time the role of the gut microbiota in a mouse model of leukemia (transplantation of BaF3 cells containing ectopic expression of Bcr-Abl), characterized at the final stage by a loss of fat mass, muscle atrophy, anorexia and inflammation. The gut microbial 16S rDNA analysis, using PCR-Denaturating Gradient Gel Electrophoresis and quantitative PCR, reveals a dysbiosis and a selective modulation of *Lactobacillus* spp. (decrease of *L. reuteri* and *L. johnsonii/gasseri* in favor of *L. murinus/animalis*) in the BaF3 mice compared to the controls. The restoration of *Lactobacillus* species by oral supplementation with *L. reuteri* 100-23 and *L. gasseri* 311476 reduced the expression of atrophy markers (Atrogin-1, MuRF1, LC3, Cathepsin L) in the gastrocnemius and in the tibialis, a phenomenon correlated with a decrease of inflammatory cytokines (interleukin-6, monocyte chemoattractant protein-1, interleukin-4, granulocyte colony-stimulating factor, quantified by multiplex immuno-assay). These positive effects are strain- and/or species-specific since *L. acidophilus* NCFM supplementation does not impact on muscle atrophy markers and systemic inflammation. Altogether, these results suggest that the gut microbiota could constitute a novel therapeutic target in the management of leukemia-associated inflammation and related disorders in the muscle.

## Introduction

The development of cancer is often accompanied by modifications of host energy homeostasis [1], [2]. Up to 50% of cancer patients suffer from adipose tissue loss and skeletal muscle atrophy – called cachexia – resulting in a reduced quality of life and in a shortened survival time [1]. The incidence of cachexia depends on cancer type [2]. 39% of patients with acute non lymphocytic leukemia experience weight loss in the six month-period preceding chemotherapy [3]. Weight loss and anorexia have also been described in acute childhood lymphoblastic leukemia and in chronic myeloid leukemia, a pathology characterized, among other things, by the fusion gene Bcr-Abl [4], [5].

The mechanisms implicated in cancer-related fat mass loss are poorly understood, but possibly imply the activation of adipose triglyceride lipase and hormone-sensitive lipase [6]. Muscle atrophy in cancer cachexia has been more extensively studied. Atrogin-1/Muscle Atrophy F-box (Mafbx) and Muscle RING Finger 1 (MuRF1), two ubiquitin ligases of the ubiquitin-proteasome pathway, are markers of skeletal muscle atrophy and are induced by cancer cachexia [7]–[9]. Overexpression of Atrogin-1 in myotubes produces atrophy, whereas mice deficient in Atrogin-1 and MurF1 are found to be resistant to atrophy [7]. MurF1 is involved in the breakdown of myofibrillar proteins (actin, myosin heavy chain), whereas, by contrast, Atrogin-1 has a role in the control of protein synthesis [10]–[12]. These markers are therefore considered as potential drug targets for the treatment of muscle atrophy [7]. The light chain 3 of microtubule-associated proteins (LC3), essential to autophagosome formation, and Cathepsin L, a lysosomal protease, are induced in muscle atrophy mouse models, i.e. amyotrophic lateral sclerosis, and might also be implied in cancer-related muscle atrophy [9], [13]. Several mechanisms have been proposed to explain the catabolic state associated with cancer in general: anorexia, futile cycles and increased thermogenesis. Furthermore, inflammatory mediators, such as interferon-gamma (IFN-γ), interleukin-6 (IL-6) and tumor necrosis factor-alpha (TNF-α) may contribute to anorexia, adipose tissue loss and muscle atrophy [1].

The gut microbiota, composed of a hundred trillion bacteria, is now considered a metabolic community, able to regulate host physiology and immunity [14], [15]. Gut microbes influence local and systemic inflammation through pattern recognition receptors [16], [17]. It is now clearly established that gut microbes may regulate fat mass expansion, e.g. through the fermentation products and the inhibition of the fasting induced adipose factor [18]–[22]. Bäckhed *et al.* also reported an increased AMPK activity in the muscle of germ-free animals [21]. Moreover, we have shown that changing the gut microbiota using prebiotics increases muscle mass in obese and type 2 diabetic mice [22]. These studies suggest a link between gut microbes and muscle metabolism. However, the gut microbes-muscle axis remains poorly explored in pathophysiological conditions, like cancer cachexia.

Intestinal dysbiosis means “alterations in the composition and/or activity of the gut microbiota in comparison to healthy individuals” and has been associated with inflammatory or metabolic diseases like inflammatory bowel disease, obesity, type 2 diabetes and necrotizing enterocolitis [23]. We have hypothesized that intestinal dysbiosis might also be associated with leukemia-related cachectic features and that disturbances in the symbiotic gut microbiota-host relationship might exist in case of systemic cancer development.

In the present study, we have used a mouse model which mimics an acute leukemia (iv injection of BaF3 cells transfected with a Bcr-Abl oncogene) [24], [25], and that is characterized by cachectic symptoms like anorexia, loss of fat mass and muscle atrophy.

## Materials and Methods

### Ethics Statement

The agreement of the animal experiments performed in this study was given by the ethical committee for animal care of the Health Sector of the Université catholique de Louvain, under the supervision of Prof. F Lemaigre et JP Dehoux under the specific number 2010/ULC/MD/022. Housing conditions were as specified by the Belgian Law of April 6, 2010, on the protection of laboratory animals (Agreement LA 1230314). All efforts were made to minimize suffering of animals.

### Cell Culture

The BaF3 cell line transfected with Bcr-Abl was a gift from Dr. K. Bhalla (MCG Cancer Center, Medical College of Georgia, Augusta, GA, USA). The BaF3 cells were maintained in RPMI1640 medium supplemented with 10% fetal bovine serum (PAA clone, PAA, Pasching, Austria), streptomycin 100 µg/ml, penicillin 100 IU/ml, and 1% of non-essential amino acids solution (Gibco, Inchinnan, Scotland) at 37°C in humidified 5% CO_2_. The generation of these cells is described in detail elsewhere [25], [26].

### Animals

Female BALB/c mice (5-week-old, Charles River, France) were housed with two mice per cage in a 12 h light/dark cycle. The intake of food and water was monitored at least 3 times a week in all experiments.

After an acclimatization period of 1 week, a saline solution or BaF3 cells (1×10^6^ cells in 0.1 ml saline) were injected in the tail vein of the anesthetized mice. In a first experiment, 16 mice (8 sham injected mice, 8 mice transplanted with BaF3 cells) were killed after 13 days. In a second experiment, 20 mice (10 sham injected mice, 10 mice transplanted with BaF3 cells) were killed after 14 days. This time frame was chosen in order to avoid animal sufferance linked to severe under-nutrition and inflammation occurring the final days preceding animal death, in accordance with the rules of the ethical committee.

For the dietary restriction experiment (third experiment), 18 mice were divided into two groups: 8 control and 10 dietary restricted mice (DR). All mice received a sham injection on day 0. From 10 days after the sham injection, DR mice received the same relative amount of diet (as a percentage of their baseline consumption) as the BaF3 mice from the first experiment described above. Food was divided in two equal portions, delivered in the morning and in the evening respectively. Mice were killed after 13 days.

In a fourth experiment, 31 mice were divided into four groups: 8 sham injected control mice, 8 sham injected mice supplemented with lactobacilli, 8 mice transplanted with BaF3 cells and 7 mice transplanted with BaF3 cells and supplemented with lactobacilli. The lactobacilli supplement was a mixture of *Lactobacillus reuteri* 100-23 and *Lactobacillus gasseri* 311476 (Institut Pasteur de Lille; cultured, frozen and quantified after thawing) administrated in the drinking water at 2×10^8^ CFU/ml of each strain, starting the first day after BaF3 inoculation. Lactobacilli solution was changed every day and contained in bottles specially designed to allow easy access to small drinking volumes. Total fat mass was determined two days before and twelve days after BaF3 inoculation with a 7.5 MHz Time domain-Nuclear magnetic resonance (TD-NMR) (LF50 minispec, Bruker, Germany). Mice were killed after 13 days.

In a fifth experiment, 35 mice were divided into four groups: 8 sham injected control mice, 9 sham injected mice supplemented with *Lactobacillus acidophilus* NCFM, 10 mice transplanted with BaF3 cells and 8 mice transplanted with BaF3 and supplemented with *L. acidophilus* NCFM. *L. acidophilus* NCFM (kindly gifted by Metagenics Belgium, Ostende, Belgium) was administrated as described in the fourth experiment. Mice were killed after 13 days.

Data from a sixth experiment (8 sham injected mice, 8 mice transplanted with BaF3 cells, killed after 13 days) were added for the correlation analyses presented in Figure S3.

### Blood and Tissue Samples

At the end of the experiments, blood glucose was determined with a glucose meter (Roche Diagnostics, Mannheim, Germany) on 3.5 µl of blood collected from the tip of the tail vein. Mice were anaesthetized with ketamine/xylazine i.p., 100 and 10 mg/kg respectively, or with isoflurane gas (Abbot, Ottignies, Belgium). Retro-orbital blood was collected in EDTA tubes, centrifuged (13000 g, 3 min) and the plasma was stored at −80°C. Mice were sacrificed using cervical dislocation. The liver was removed and weighed. A piece of the liver tissue was fixed in formalin for further histological analysis, and the remaining liver was clamped in liquid N_2_ and stored at −80°C. The spleen, adipose tissues, muscles and cecum content were collected, weighed and frozen in liquid N_2_.

### Tissue mRNA Analyses

Total RNA was isolated from tissues using the TriPure isolation reagent kit (Roche Diagnostics, Penzberg, Germany). cDNA was prepared by reverse transcription of 1 µg total RNA using the Kit Reverse transcription System (Promega, Madison, WI). Real-time polymerase chain reaction (PCR) was performed with a StepOnePlus Real-Time PCR System and software (Applied Biosystems, Den Ijssel, The Netherlands) using SYBR Green (Applied Biosystems and Eurogentec, Verviers, Belgium) for detection. All samples were run in duplicate in a single 96-well reaction plate, and data were analyzed according to the 2^− ΔΔCT^ method. The purity of the amplified product was verified by analyzing the melt curve performed at the end of amplification. The ribosomal protein L19 (RPL19) gene was chosen as a reference gene. The primer sequences for the targeted mouse genes are detailed in Table S1.

### Blood Analyses

Spectrophotometric kits were used to determine plasma triglycerides and cholesterol concentrations (Diasys, Sopachem, Brussels, Belgium). Plasma interleukin-4 (IL-4), interleukin-10 (IL-10), interleukin-6 (IL-6), monocyte chemoattractant protein-1 (MCP-1), interleukin-8 (IL-8) and granulocyte colony-stimulating factor (G-CSF) were measured using a customized multiplex kit (Bio-Rad, Nazareth, Belgium) with the Luminex technology (Bio-Plex, Bio-Rad).

### Gut Microbiota Analyses

The gut microbiota composition was assessed by 16S rRNA gene analyses using PCR followed by denaturing gradient gel electrophoresis (DGGE), and quantitative polymerase chain reaction.

Genomic DNA was extracted from the cecal content using a QIAamp DNA Stool Mini Kit (Qiagen, Hilden, Germany) according to the manufacturer’s instructions. Denaturing Gradient Gel Electrophoresis (DGGE) was performed on the total bacteria amplicons that were generated with primers targeting the V3 region of bacterial 16S rRNA gene [27]. The sequences are reported in Table S1. The PCR mixture (50 µl) contained 5 µl of 10× NH_4_ reaction buffer; 2.5 µl of MgCl_2_ (50 mM, 0.1 mg/ml); 2.5 µl of a deoxynucleoside triphosphate preparation (containing each nucleotide at a 2 mM concentration); 1 µl of each primer (10 µM); 0.25 µl of Taq polymerase (5 U/µl); 36.75 µl of distilled water and 1 µl of DNA. The PCR program was used as follows: an initial denaturation at 94°C for 5 min; 30 cycles of denaturation at 94°C for 20 s, annealing at 55°C for 45 s, and extension at 72°C for 1 min; and a final extension at 72°C for 7 min, followed by cooling to 4°C. The PCR was verified by mixing 10 µl of the PCR product with 2 µl of a loading dye and electrophoresing it on a 1.5% (wt/vol) agarose gel for 15 min at 120 V flanked by a 1 kb DNA ladder (Promega, Madison, WI). The PCR products were analyzed on a 35% to 60% DGGE gel using a protocol described by Muyzer *et al.* [27] and modified as described below. Electrophoresis was performed for 14 h at 85 V in 1× TAE buffer at a constant 60°C temperature using a Dcode system (Model DGGE-2001, CBS Scientific Company). The gel was stained with SYBR Gold (20 µl of SYBR Gold in 250 ml of 1× TAE buffer) for 30 min; this was followed by examining the DGGE band profiles under a UV light. Digital image capturing was performed using a UVtec UVIdoc gel documentation system. The DGGE fingerprints obtained were analyzed using FPQuest software (Bio-Rad, Hercules, CA).

Quantitative PCR was performed as described in “Tissue mRNA analyses”. To detect some *Lactobacillus* spp. (*Lactobacillus* spp. [I] in the first experiment, third experiment), *Bifidobacterium* spp. and *Bacteroides* spp., we used probes and a TaqMan Universal PCR Master Mix, as described previously [28]. The primers and probes are detailed in Table S1. The cycle threshold of each sample was then compared with a standard curve (performed in triplicate) made by diluting genomic DNA (five-fold serial dilution) (BCCM/LMG, Ghent, Belgium and DSMZ, Braunshweig, Germany). Prior to isolating the DNA, the cell counts were determined in culture and expressed as “colony-forming unit” (CFU). For *L. johnsonii/gasseri*, *L. reuteri* and *L. murinus/animalis*, standard DNA was quantified based on *L. acidophilus* DNA.

### Statistical Analyses

Results are presented as the mean ± SEM. Statistical significance of differences between groups was assessed either by a Student’s t-test when comparing 2 groups or by one-way ANOVA followed by post hoc Tukey’s multiple comparison test when comparing 3 groups or more. Correlations between parameters were assessed by the Pearson’s test. The food intake evolution was analysed by two-way ANOVA followed by Bonferroni post hoc test. P<0.05 was considered statistically significant. All analyses were performed with GraphPad Prism 5.0 (GraphPad Software, San Diego, CA), except for the principal component analysis, performed with R [29].

## Results

### Mice Transplanted with BaF3 Cells (BaF3 mice) Present Cancer Cell Infiltration in the Liver and the Spleen, and Develop Muscle Atrophy and Fat Loss

**Figure 1:**
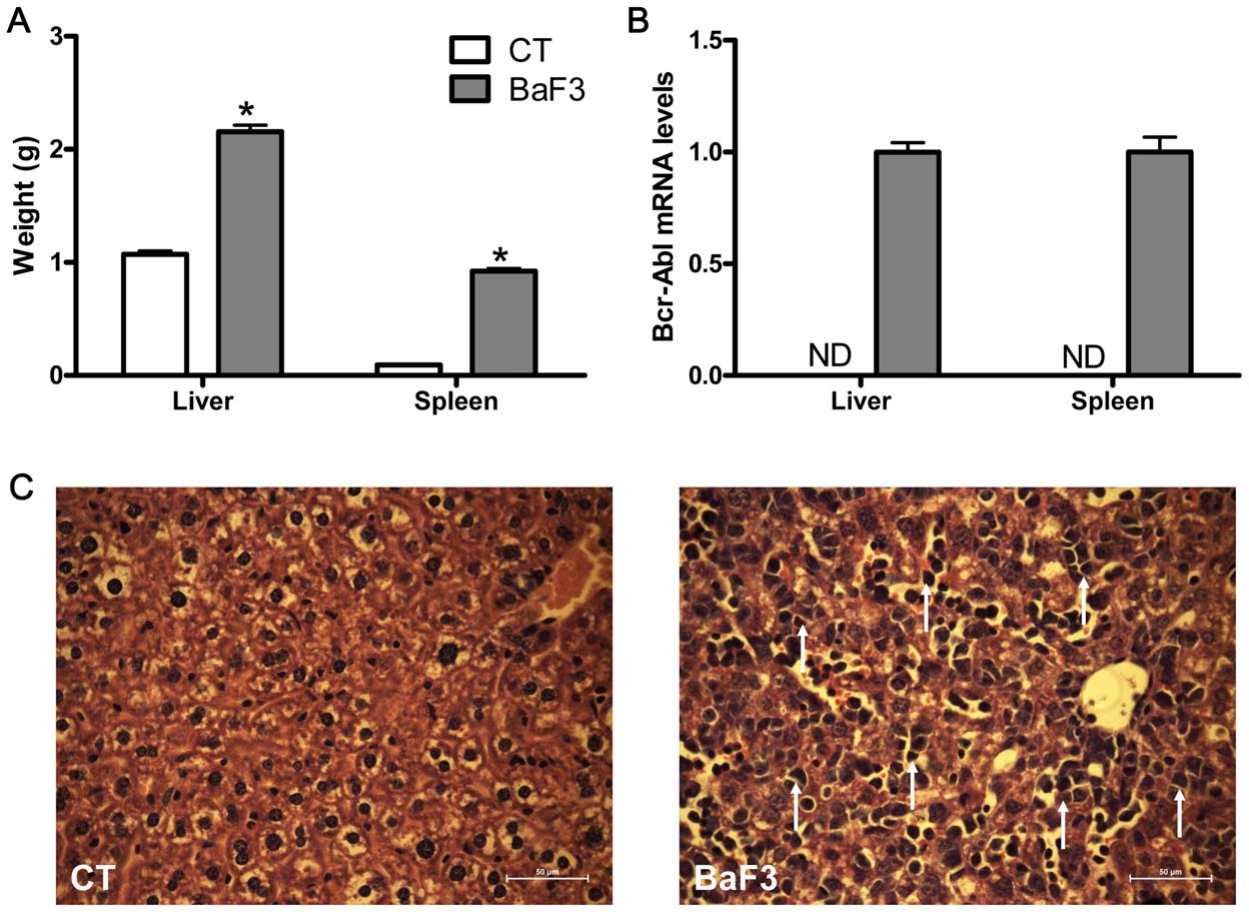
Transformed mouse proB BaF3 cells infiltrate the liver and spleen. **A**. Liver and spleen weights. N = 8. *p<0.05 vs. control. **B**. Bcr-Abl mRNA levels in liver and spleen, ND: not detected. **C.** Histological analysis of liver tissue (hematoxylin-eosin staining). Bar  = 50 µm. White arrows indicate BaF3 cells.

Mouse pro-B BaF3 cells containing ectopic expression of Bcr-Abl cells were transplanted into mice resulting in an aggressive malignancy that mimicked acute leukemia, which was characterized by an accumulation of BaF3 cells in the liver and spleen. Leukemia progression was evaluated based on the liver and spleen weight increase, Bcr-Abl mRNA expression in both organs and histological analysis of the liver parenchyma (Figure 1).

BaF3 mice exhibited a reduced food intake from 10 days after the transplantation (Figure S1A, B). Mice were sacrificed 13 days after the transplantation. BaF3 transplantation decreased adipose tissues weight (subcutaneous adipose tissue: CT: 305±20 mg; BaF3∶222±20 mg; n = 8, p = 0,01 and ovarian adipose tissue: CT: 257±21 mg; BaF3∶133±16 mg; n = 8, p<0,001). The atrophy marker expression, namely Atrogin-1, MuRF1, LC3 and Cathepsin L, was induced in the gastrocnemius muscle of BaF3 mice by 1.3-fold to 5-fold after 13 days without any changes in muscle mass (Table 1). After 14 days, the atrophy marker expression was further induced and muscle mass loss appeared significant. We thus concluded, in accordance with previously reported *in vivo* kinetics [8], [30], that Atrogin-1, MuRF1, LC3 and Cathepsin L are valuable early markers of muscle atrophy in this mouse model of leukemia.

**Table 1:** Tissue weight and atrophy marker expression in the gastrocnemius muscle.

	13 days		14 days	
	Control mice	BaF3 mice	Control mice	BaF3 mice
***Tissue weight***				
Tibialis (mg)	31±1	31±1	31±1	25±1*
Gastrocnemius (mg)	96±1	95±1	98±1	80±1*
***Markers of muscle atrophy (mRNA expression)***				
Atrogin-1	1.00±0.10	4.96±0.78*	1.00±0.09	6.98±0.99*
MuRF1	1.00±0.06	1.63±0.25*	1.00±0.05	6.83±0.70*
LC3	1.00±0.03	1.33±0.07*	1.00±0.03	1.72±0.10*
Cathepsin L	1.00±0.03	1.94±0.27*	1.00±0.04	5.07±0.43*

N = 8–10,

* p<0.05 versus control mice.

### BaF3 Mice are Characterized by Changes in Gut Microbiota Composition Occurring Regardless of the Food Intake

**Figure 2:**
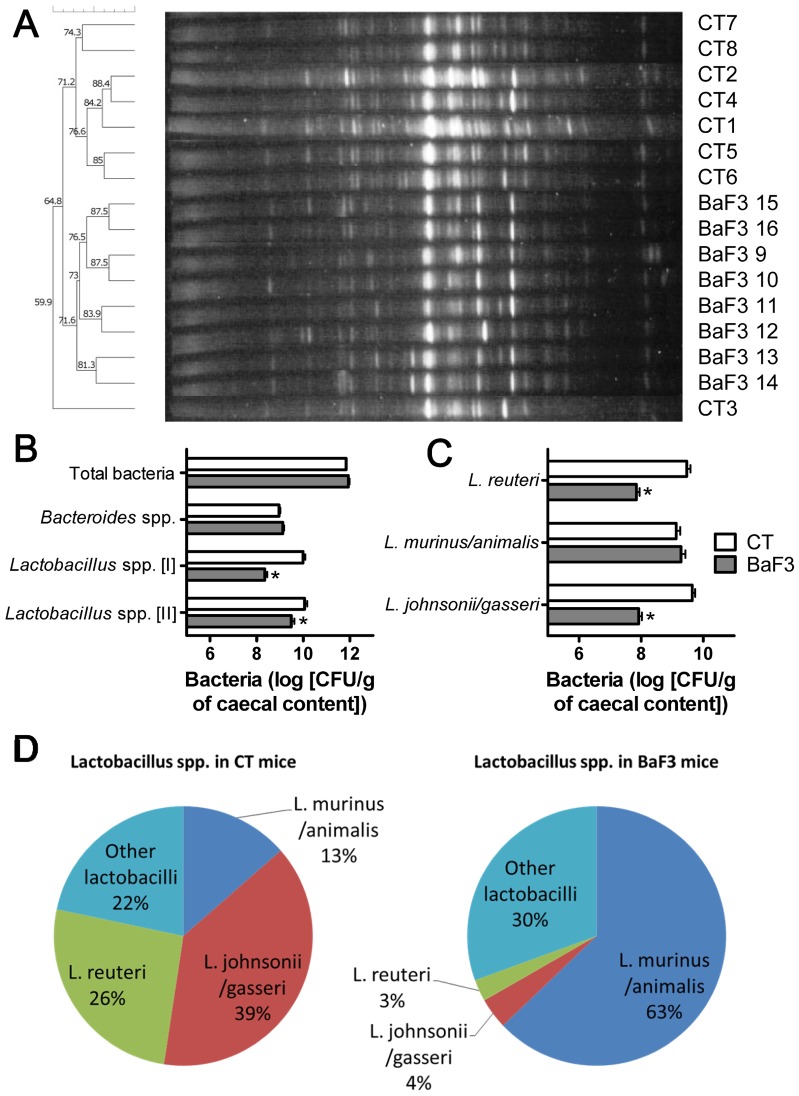
Changes in gut microbiota composition occur in mice transplanted with BaF3 cells. **A**. DGGE profiles of the bacterial DNA isolated from the cecal content. Each profile corresponds to one animal. UPGMA dendogram with Dice coefficient. **B**. Levels of total bacteria, *Bacteroides* spp. and *Lactobacillus* spp. (quantified with two different sets of primers). **C**. *Lactobacillus johnsonii/gasseri, Lactobacillus murinus/animalis* and *Lactobacillus reuteri* levels. **D**. Relative proportions of each *Lactobacillus* species, expressed as a percentage of the *Lactobacillus* spp. levels (second primer set). N = 8, *p<0.05 vs. control. Percentages of each species of lactobacilli are significantly different (p<0.05 with Student’s t-test) between control mice (CT) and mice transplanted with BaF3 cells (BaF3 mice).

To assess the presence of intestinal dysbiosis, the gut microbial composition was assessed by PCR-denaturing gradient gel electrophoresis (DGGE) and by qPCR of 16S rRNA gene sequences for total bacteria, *Lactobacillus* spp., *Bifidobacterium* spp. (two Gram-positive genera known for their anti-inflammatory properties [31], [32]) and *Bacteroides* spp. (a predominant Gram-negative genus). The DGGE fingerprints for total bacteria revealed two separate clusters of mice, with the BaF3 mice clustering separately from the control mice (Figure 2A). Total bacteria and *Bacteroides* spp. were present at similar levels in the cecal content of the control mice and the BaF3 mice, whereas the lactobacilli levels were reduced in the BaF3 mice versus the control mice (Figure 2B). The lack of bifidobacteria (data not shown) appears to be a distinctive feature of young BALB/c mice [33].

As the melt curve shape difference suggested a change in the proportions of the species within the *Lactobacillus* genus (Figure S2), the common murine *Lactobacillus* species were enumerated by qPCR using specific species-targeting primers. *Lactobacillus johnsonii/gasseri* and *Lactobacillus reuteri* were significantly decreased whereas *Lactobacillus murinus/animalis* were not modified (Figure 2C). The level of each of these species could also be expressed as a percentage of the total *Lactobacillus* population (Figure 2D); the 3 species comprised 70 to 79% of the lactobacilli present. Expressed in percentage, the relative ratio of *Lactobacillus* species found in the BaF3 mice was modified in favor of *L. murinus/animalis* at the expense of *L. johnsonii/gasseri* and *L. reuteri*. *L. acidophilus* and *L. helveticus* were also tested but represented less than 1% of the lactobacilli present (data not shown). Interestingly, a highly significant negative correlation was found between the level of lactobacilli in the cecal content and the atrophy marker expression in the gastrocnemius muscle (Atrogin-1, MurF1, LC3 and Cathepsin L) (Figure S3).

The decreased food intake observed at the end of the experiment could theoretically contribute to the decreased level of *Lactobacillus* spp., e.g. through the changes of substrates available for bacterial fermentation. To explore this hypothesis, a separate set of experiments was performed. Mice were dietary restricted for the last three days, as demonstrated in the food intake graph (Figure S1A, B). No differences in the cecal levels of *Bacteroides* spp., total lactobacilli or specific lactobacilli species were observed between dietary-restricted and *ad libitum*-fed mice (Figure S1C). This set of data thus demonstrate that intestinal dysbiosis occurs in leukemic mice, independently of the reduced food intake.

### Supplementation with *Lactobacillus reuteri* 100-23 and *Lactobacillus gasseri* 311476 Decreases Pro-inflammatory Cytokines and the Expression of Muscle Atrophy Markers

**Figure 3:**
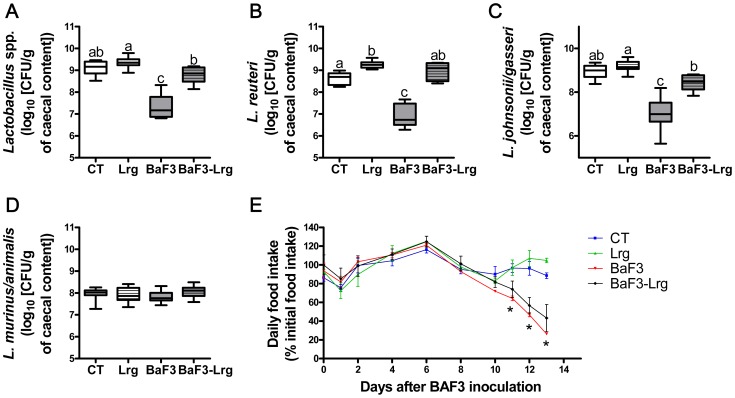
Supplementation with *Lactobacillus reuteri* 100-23 and *Lactobacillus gasseri* 311476 mixture restores the lactobacilli levels. **A–D**. *Lactobacillus* spp., *L. reuteri*, *L. jonhsonii/gasseri* and *L. murinus/animalis* levels in control mice (CT), in mice receiving lactobacilli (Lrg), in mice transplanted with BaF3 cells (BaF3) and in mice transplanted with BaF3 cells and receiving lactobacilli (BaF3-Lrg). **E**. Daily food intake. N = 7–8. Data with different superscript letters are significantly different (p<0.05). For the food intake, n = 4 and *p<0,05 BaF3 and BaF3-Lrg mice versus CT and Lrg mice.

To evaluate the relevance of the drop in lactobacilli to the cachectic features occurring in the BaF3 mice, we set up an *in vivo* experiment consisting of a supplementation with two *Lactobacillus* strains. *Lactobacillus reuteri* 100-23 and *Lactobacillus gasseri* 311476 were chosen because they are members of the predominant lactobacilli species that were decreased in the BaF3 mice, namely *Lactobacillus reuteri* and *Lactobacillus johnsonii/gasseri.* Moreover, one of these two strains (*L. reuteri* 100-23) exhibits immunoregulatory capacities [34]. Supplementation with a mixture of both strains (2×10^8^ CFU/ml of each strain) restored the *Lactobacillus* spp. levels in BaF3 mice, both at the genus and at the species level, without changing *L. murinus* count (Figure 3A–D). The daily food intake was unchanged upon lactobacilli supplementation (Figure 3E). Lactobacilli supplementation did not impact on either body weight gain, nor on leukemia progression (similar liver and spleen weight, and expression of Bcr-Abl in the liver) (Table 2). Lactobacilli did not counteract the fat mass loss assessed using TD-NMR and by weighing adipose fat pads. We also measured blood glucose and lipid parameters. Glycemia and serum cholesterol were decreased in the BaF3 mice supplemented or not with lactobacilli. Serum triglycerides were not affected by the presence of leukemia, or by lactobacilli supplementation.

**Table 2:** Body weight gain, leukemia- and fat mass-related parameters, blood glucose and lipids.

	Control	Lrg	BaF3	BaF3-Lrg
Body weight gain (g)	1.67±0.32	2.00±0.18	1.65±0.19	1.51±0.30
Liver weight (g)	0.97±0.03^a^	1.00±0.03^a^	2.31±0.04^b^	2.07±0.13^b^
Spleen weight (g)	0.08±0.01^a^	0.08±0.01^a^	0.85±0.02^b^	0.84±0.04^b^
Hepatic Bcr-Abl mRNA levels (relative expression)	ND	ND	1.00±0.10	1.07±0.13
Change in fat mass (g)	0.50±0.09^a^	0.58±0.18^a^	−1.35±0.09^b^	−0.93±0.15^b^
Ovarian adipose tissue (g)	0.26±0.02^a^	0.26±0.02^a^	0.12±0.01^b^	0.13±0.01^b^
Subcutanous adipose tissue (g)	0.30±0.02^a^	0.33±0.02^a^	0.20±0.01^b^	0.20±0.01^b^
Glycemia (mM)	5.42±0.20^ab^	5.67±0.18^b^	4.42±0.35^c^	4.58±0.23^ac^
Plasma cholesterol (mM)	1.60±0.06^a^	1.61±0.05^a^	0.96±0.06^b^	1.07±0.09^b^
Plasma triglycerides (mM)	1.46±0.16	1.58±0.14	1.31±0.13	1.05±0.09

N = 7–8, data with different superscript letters are significantly different (p<0.05). ND  =  not detected.

**Figure 4:**
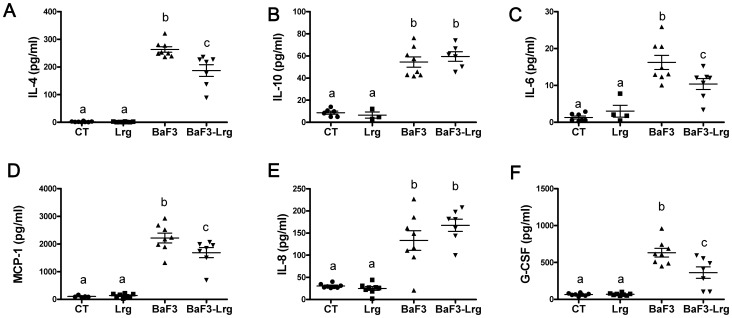
Supplementation with *Lactobacillus reuteri* 100-23 and *Lactobacillus gasseri* 311476 mixture reduces inflammatory cytokines. **A–F**. Plasma levels of interleukin 4 (IL-4), interleukin 10 (IL-10), interleukin 6 (IL-6), monocyte chemoattractant protein 1 (MCP-1), interleukin 8 (IL-8), and granulocyte colony-stimulating factor (G-CSF) in control mice (CT), in mice receiving lactobacilli (Lrg), in mice transplanted with BaF3 cells (BaF3) and in mice transplanted with BaF3 cells and receiving lactobacilli (BaF3-Lrg). Data with different superscript letters are significantly different (p<0.05).

Knowing the role of some lactobacilli in the control of innate immunity and inflammation, we quantified the relevant inflammatory markers in mice sera. Interleukin 4 (IL-4), interleukin 10 (IL-10), interleukin 6 (IL-6), monocyte chemo-attractant protein 1 (MCP-1), interleukin 8 (IL-8), and granulocyte colony-stimulating factor (G-CSF) levels were all increased in the BaF3 mice compared to the control mice (Figure 4A–F). Interestingly, lactobacilli supplementation reduced the level of IL-4, MCP-1, G-CSF and IL-6 in the BaF3 mice. IL-10 and IL-8 remained at a similar elevated level in the BaF3 mice with or without lactobacilli.

**Figure 5:**
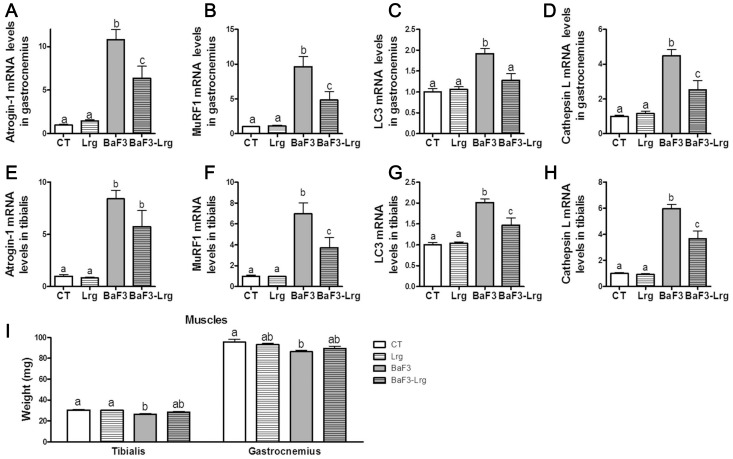
Supplementation with *Lactobacillus reuteri* 100-23 and *Lactobacillus gasseri* 311476 mixture reduces muscle atrophy markers. **A–D**. Atrophy marker expression (Atrogin-1, MurF1, LC3 and Cathepsin L) measured in the gastrocnemius muscle of control mice (CT), mice receiving lactobacilli (Lrg), mice transplanted with BaF3 cells (BaF3) and mice transplanted with BaF3 cells and receiving lactobacilli (BaF3-Lrg). **E–H**. Atrophy marker expression (Atrogin-1, MurF1, LC3 and Cathepsin L) measured in the tibialis muscle. **F**. Muscle weights. N = 7–8. Data with different superscript letters are significantly different (p<0.05).

The increased production of IL-6 has been implicated in B-cell malignancies and could play a role in human and rodent cachexia, as suggested before by results obtained with anti-IL-6 mAb therapy [35]. Inflammatory mediators have been reported to potentially contribute to anorexia and muscle atrophy [1]. Since lactobacilli supplementation decreased IL-6 serum levels, we analyzed muscle atrophy markers. Supplementation with *L. reuteri* 100-23 and *L. gasseri* 311476 lowered the induction of muscle atrophy markers (Atrogin-1, MuRF1, LC3 and Cathepsin L) in the gastrocnemius muscle (Figure 5A–D). The induction of atrophy markers in the tibialis muscle was also reduced by the lactobacilli supplementation (although not significantly for Atrogin-1, Figure 5E–H). Lactobacilli supplementation did not significantly modify gastrocnemius weight but increased by 8% tibialis muscle weight (p = 0.05, t-test BaF3 versus BaF3-Lrg) (Figure 5I).

A principal component analysis (PCA), taking into account all lactobacilli-, atrophy- and inflammation-related parameters, shows no difference between the control mice and the mice supplemented with lactobacilli (p = 0.42, t-test on dimension 1), whereas the BaF3 mice supplemented or not with lactobacilli constitute two separate groups (p = 0.0051, t-test on dimension 1) (Figure S4), further demonstrating that lactobacilli supplementation impacts on atrophy- and inflammation-related parameters.

Furthermore, the 4 atrophy markers in the gastrocnemius muscle and the 6 plasma inflammatory proteins are positively correlated (e.g. MuRF1 expression and serum IL-6: r = 0.81, p<0.0001, n = 31), whereas *Lactobacillus* spp. level is negatively correlated with the 4 muscle atrophy markers, assessed in the gastrocnemius muscle, and the 6 plasma inflammatory proteins (e.g. *Lactobacillus* spp. and LC3 expression: r = −0.84, p<0.001, n = 31).

### The Lactobacilli Effect on Inflammation and Muscle Atrophy in the BaF3 Mice is Species- or Strain-specific

As beneficial properties of lactobacilli are known to be strain-specific [36], we also evaluated the effect of *Lactobacillus acidophilus* NCFM, a well-known and widely used probiotic strain [37]. Supplementation with *L. acidophilus* NCFM restored the *Lactobacillus* spp. level in the cecal content of the BaF3 mice to the values observed in the control mice (Figure S5A). *L. acidophilus* NCFM supplementation had no impact on the liver and spleen weights, and did not modify markers of muscle atrophy (Atrogin-1 and Cathepsin L) (Figure S5B-D). Consistently, no modification of plasma inflammatory markers (IL-4, IL-10, IL-6, MCP-1, IL-8, G-CSF) were noticed between the BaF3 mice and the BaF3 mice supplemented with *L. acidophilus* NCFM (Figure 5E–J).

## Discussion

Nowadays, the gut microbiota is considered a salient regulator of key host functions (energy metabolism, immunity, endocrine function) in several physiological and pathological conditions [16], [38], [39]. To our knowledge, its role in the occurrence of cachectic features associated with leukemia progression has never been investigated. In this study, we bring experimental evidence supporting the concept that the gut microbiota interacts with host tissues to control leukemia-related inflammation and induction of muscle atrophy markers. In fact, we propose that, in an acute leukemia mouse model, i) dysbiosis occurs, ii) a lower level of specific lactobacilli in the gut is associated with muscle atrophy markers and pro-inflammatory processes occurring upon leukemia progression and iii) counteracting the drop in the level of the *Lactobacillus reuteri* and *gasseri/johnsonii* using a selected probiotic approach reduces serum pro-inflammatory cytokines and decreases muscle atrophy marker expression.

We show here for the first time that the gut microbiota composition is modified during leukemia progression. In this mouse model, *Lactobacillus* spp. levels decrease by a factor of fifty, affecting specific lactobacilli species such as *L. johnsonii/gasseri*, or *L. reuteri*, whereas other groups remain unchanged (*L. murinus/animalis*). Such specific changes inside the *Lactobacillus* genus have previously been reported in a genetic mouse model of colitis [40]. The authors suggested as a mechanism a change in the availability of a *L. reuteri* specific substrate.

Crawford *et al.* demonstrated that a 24-hour starvation period was enough to significantly modify the mouse gut microbiota composition in favor of an increased proportional representation of the *Bacteroidetes* and a decrease in *Firmicutes* [41]. Therefore, we performed a dietary restriction experiment which allowed us to conclude that the decreased lactobacilli levels could not be explained by the decreased food intake, but were clearly linked to another phenomenon, associated with the progression of the disease.

To counteract the reduced numbers of lactobacilli, and to assess the relevance of this reduction on systemic alterations occurring in leukemic mice, we performed a probiotic approach consisting in the oral administration of two live lactobacilli. We used strains which belong to the species whose levels were decreased in the BaF3 mice, *L. reuteri* 100-23 and *L.gasseri* 311476. Interestingly, *L. reuteri* and *L. gasseri* restoration decreased the plasma level of inflammatory cytokines (IL-4, MCP-1, IL-6, G-CSF) and maintained the plasma level of the anti-inflammatory IL-10. The ability of specific *Lactobacillus* strains, including *L. reuteri* 100-23, to modulate inflammation and systemic immunity is now clearly established [34], [36], [42], [43]. Lactobacilli counts were reduced in leukemic mice and this decrease correlated negatively with the atrophy markers in the muscle. Pro-inflammatory cytokines, such as IL-6 or TNF-α, have been proposed to contribute to muscle atrophy [1]. Of note, experiments revealed that TNF-α level was not increased in the plasma of BaF3 mice (data not shown). IL-4 is the most powerful cytokine that can initiate T helper 2 cell differentiation [44]. The anti-inflammatory effect of the selected lactobacilli, including decreased Th2 immunity, could contribute to the reduction in atrophy marker expression in both gastrocnemius and tibialis muscles. This reduction in the muscle atrophy marker expression could be of interest in therapeutics. Indeed, reducing atrophy markers, *in vivo* or *in vitro*, directly or indirectly, attenuates muscle atrophy [45]–[47]. This effect, however, might be strain- and/or species-specific since it was not observed with e.g. *Lactobacillus acidophilus* NCFM. Because beneficial properties of lactobacilli are known to be strain-specific [36], we hypothesize that the inflammatory properties of the strain could constitute a rational argument for the probiotics selection for the future experiments.

Lipopolysaccharides (LPS) are pro-inflammatory compounds of bacterial origin [48]. They are able to induce muscle atrophy *in vivo* and *in vitro* [49] and are increased in the serum of acute leukemic patients [50]. In the present study, we did not find significant modification in serum LPS (data not shown). This suggests that the pro-inflammatory process is not dependent, in this model, on LPS translocation across the gut barrier.

Concerning the cancer progression itself, inflammatory mediators such as cytokines and chemokines could in some cases play opposite roles on cancer progression through their action on survival pathways and metastasis. Generally, inflammation is considered as pro-tumorigenic [51], [52]. An anti-inflammatory approach thus arouses full interest in the field of cancer therapy [51]. In our model, decreasing inflammation through specific lactobacilli supplementation did not impact on leukemia progression, but seemed to selectively target the muscle tissue by decreasing the muscle atrophy marker expression.

In conclusion, we highlighted that the gut microbiota composition is modified in a mouse model which mimics acute leukemia. Moreover, we illustrate that restoring the *Lactobacillus* levels by adding specific strains reduces the inflammation, an effect correlated with the improvement of skeletal muscle atrophy markers. Our data open a new door to understanding the mechanisms underlying the gut microbiota-host interplay in the context of systemic cancers. Obviously, the role of the gut microbiota in systemic immunity and related muscle disorders, as well as the existence of intestinal dysbiosis, warrants further investigations in leukemic patients, before proposing novel therapeutic approaches.

## Supporting Information

Figure S1 **The decreased food intake observed at the end of the experiment is not responsible for the decreased** ***Lactobacillus*** **spp. levels.** **A**. Daily food intake of mice that received a transplant of BaF3 cells (BaF3) and their control (CT1); and of dietary restricted (DR) mice and their control (CT2). N = 4–5. **B**. Total food intake from day 11 to day 13, N = 4–5. **C**. *Bacteroides* spp., *Lactobacillus* spp., *L. johnsonii/gasseri* and *L. reuteri* levels. N = 8–10. *p<0.05 BaF3 vs. CT1, # p<0.05 DR vs. CT2.
(TIFF)

Figure S2 **The melt curve shape difference between the control mice and the BaF3 mice suggests that the equilibrium inside the** ***Lactobacillus*** **genus is modified.** **A, B**. Melt curves in derivative form of the PCR amplicons generated with the *Lactobacillus* spp. [II] primers. Different peaks can be assumed to represent differences in % G+C content of the amplicons (Louis P, Young P, Holtrop G, Flint HJ. (2010). Diversity of human colonic butyrate-producing bacteria revealed by analysis of the butyryl-CoA:acetate CoA-transferase gene. Environ. Microbiol. 12∶304–314.).
(TIFF)

Figure S3 ***Lactobacillus*** **spp. levels are highly correlated with muscle atrophy markers.** **A–D**. Correlations between *Lactobacillus* spp. levels and atrophy marker expression (Atrogin-1, MuRF1, LC3 and Cathepsin L) measured in the gastrocnemius muscle. Closed circle for control mice (n = 16); cross for mice transplanted with BaF3 cells (n = 15–16). The graphs are the result of two independent *in vivo* experiments pooled together. Insets indicate the Pearson correlation coefficient and the corresponding p-value.
(TIFF)

Figure S4 **Unsupervised analysis of the supplementation with** ***Lactobacillus reuteri*** **100-23 and** ***Lactobacillus gasseri*** **311476.** The principal component analysis takes into account lactobacilli levels, muscle weight, muscle atrophy marker expression (tibialis and gastrocnemius), and plasma inflammatory markers. CT  =  control mice; Lrg  =  mice receiving lactobacilli; BaF3 =  mice transplanted with BaF3 cells; BaF3-Lrg  =  mice transplanted with BaF3 cells and receiving lactobacilli.
(TIFF)

Figure S5 ***Lactobacillus acidophilus*** **NCFM supplementation does not blunt cachexia.** **A**. *Lactobacillus* spp. levels in control mice (CT), in mice receiving *L. acidophilus* NCFM (Lac), in mice transplanted with BaF3 cells (BaF3) and in mice transplanted with BaF3 cells mice and receiving *L. acidophilus* NCFM (BaF3-Lac). **B**. Liver and spleen weight. **C–D**. Atrophy marker expression (Atrogin-1 and Cathepsin L) measured in the gastrocnemius muscle. **E–J**. Plasma levels of interleukin 4 (IL-4), interleukin 10 (IL-10), interleukin 6 (IL-6), monocyte chemoattractant protein 1 (MCP-1), interleukin 8 (IL-8) and granulocyte colony-stimulating factor (G-CSF). N = 8–10. Data with different superscript letters are significantly different (p<0.05).
(TIFF)

Table S1 **Primers and probes sequences used for real-time quantitative PCR and PCR amplification before DGGE.** Ta: PCR annealing temperature (°C).
(XLS)
