# Supplementary figures and images for: Restoring Specific Lactobacilli Levels Decreases Inflammation and Muscle Atrophy Markers in an Acute Leukemia Mouse Model
Source: PLoS One. 2012 Jun 27;7(6):e37971. doi: 10.1371/journal.pone.0037971 (PMC3384645; doi:10.1371/journal.pone.0037971)

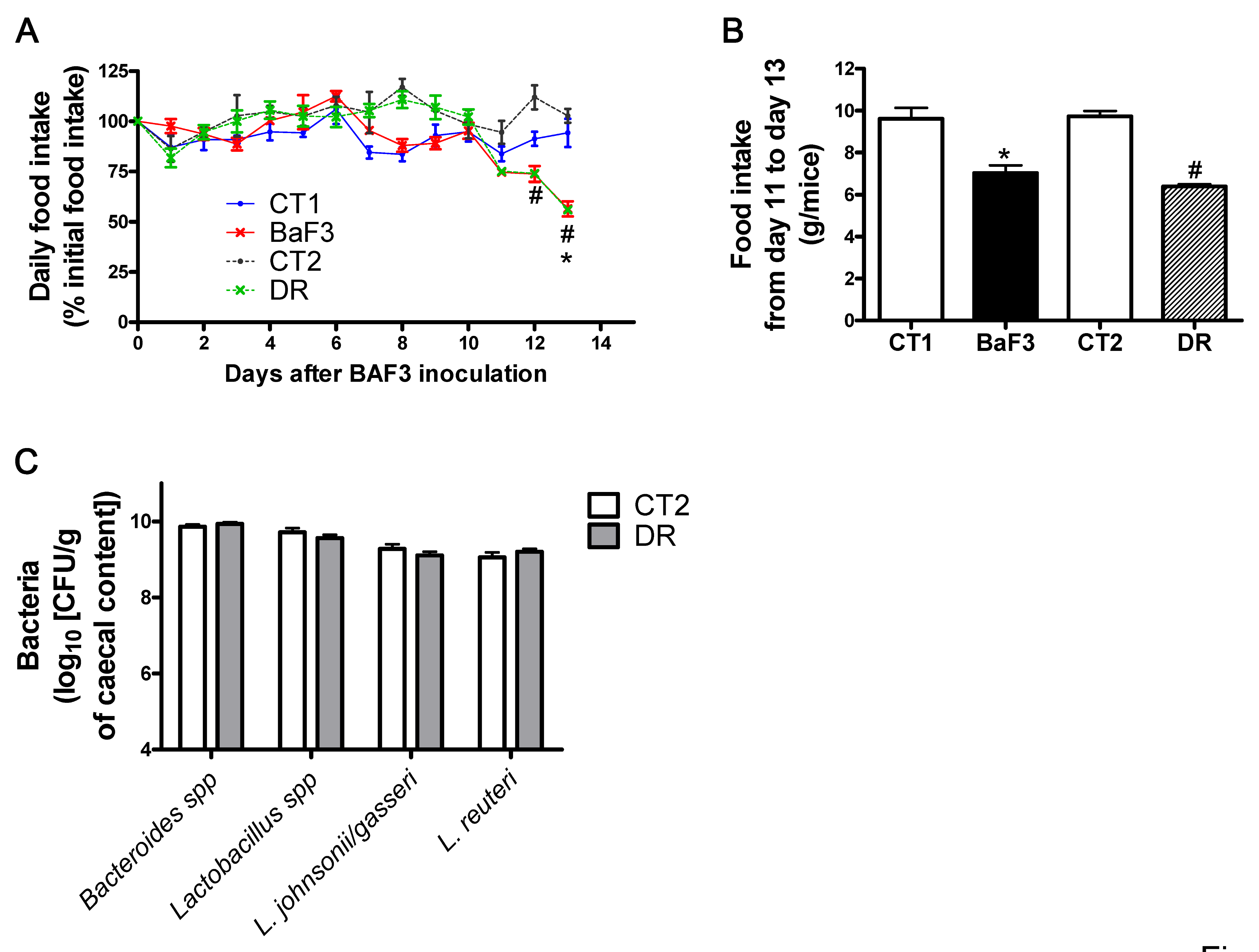

Supplement: Figure S1 — The decreased food intake observed at the end of the experiment is not responsible for the decreased Lactobacillus spp. levels. A. Daily food intake of mice that received a transplant of BaF3 cells (BaF3) and their control (CT1); and of dietary restricted (DR) mice and their control (CT2). N = 4–5. B. Total food intake from day 11 to day 13, N = 4–5. C. Bacteroides spp., Lactobacillus spp., L. johnsonii/gasseri and L. reuteri levels. N = 8–10. *p<0.05 BaF3 vs. CT1, # p<0.05 DR vs. CT2. (TIFF) [file pone.0037971.s001.tif]

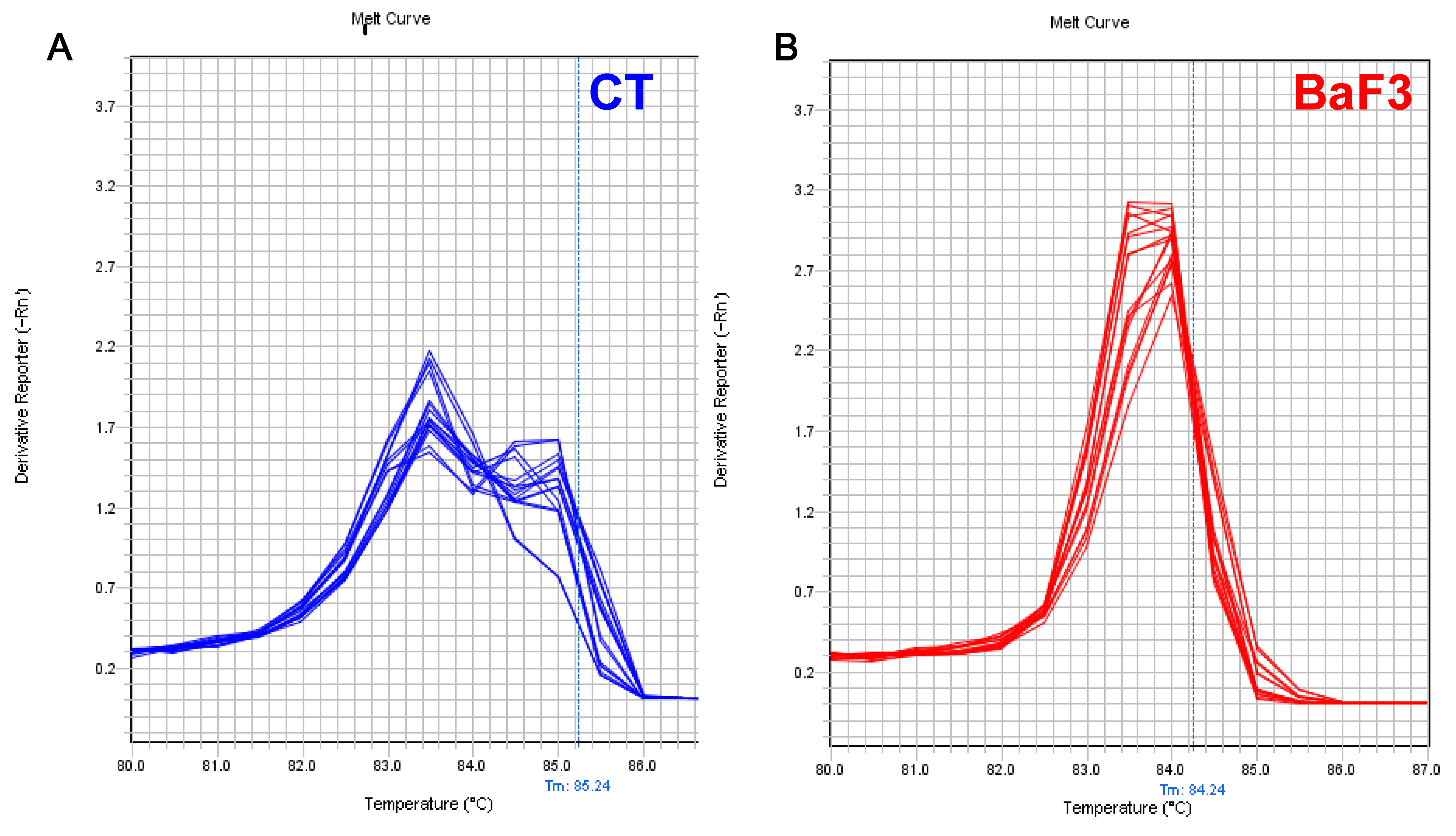

Supplement: Figure S2 — The melt curve shape difference between the control mice and the BaF3 mice suggests that the equilibrium inside the Lactobacillus genus is modified. A, B. Melt curves in derivative form of the PCR amplicons generated with the Lactobacillus spp. [II] primers. Different peaks can be assumed to represent differences in % G+C content of the amplicons (Louis P, Young P, Holtrop G, Flint HJ. (2010). Diversity of human colonic butyrate-producing bacteria revealed by analysis of the butyryl-CoA:acetate CoA-transferase gene. Environ. Microbiol. 12∶304–314.). (TIFF) [file pone.0037971.s002.tif]

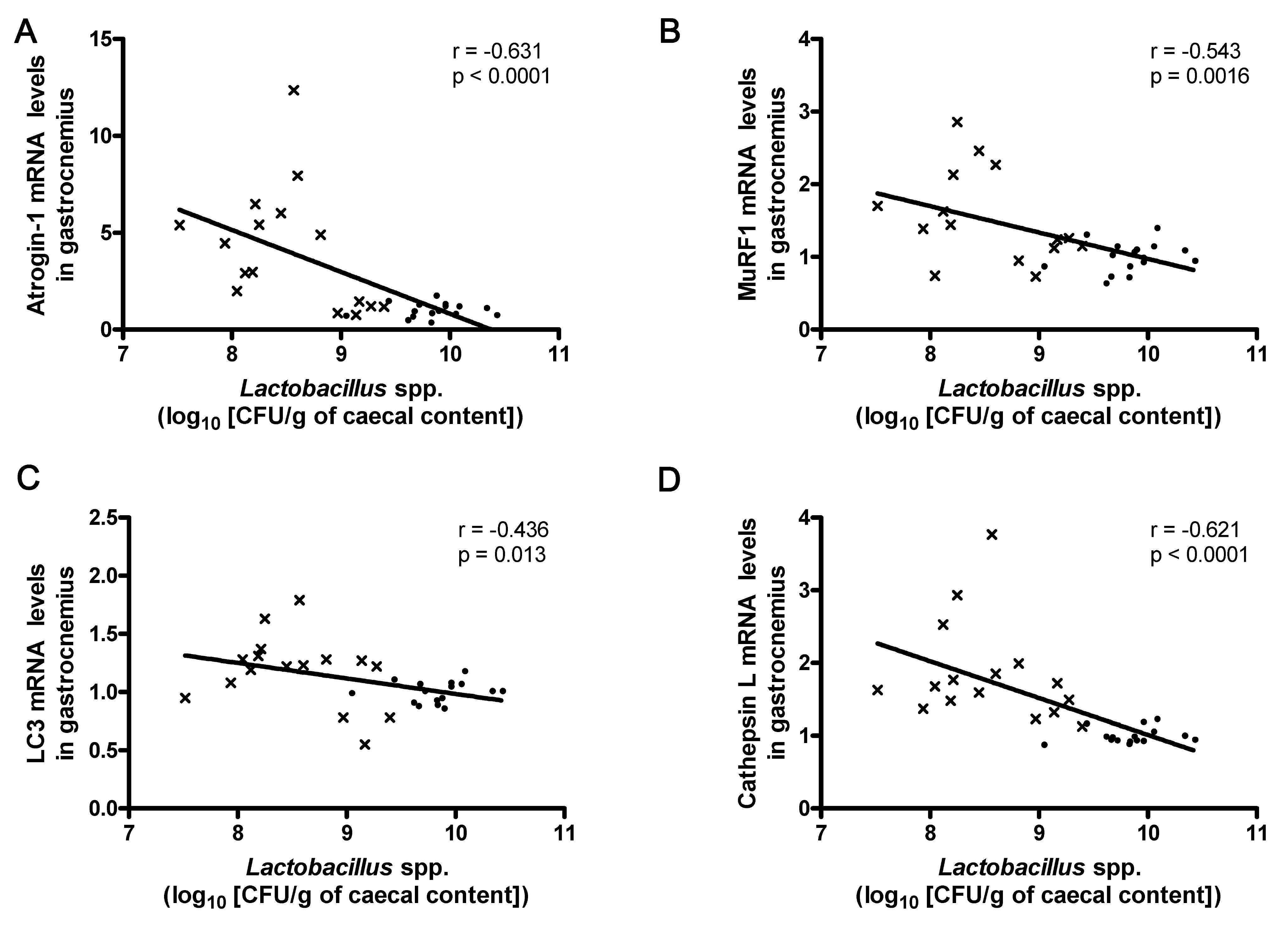

Supplement: Figure S3 — Lactobacillus spp. levels are highly correlated with muscle atrophy markers. A–D. Correlations between Lactobacillus spp. levels and atrophy marker expression (Atrogin-1, MuRF1, LC3 and Cathepsin L) measured in the gastrocnemius muscle. Closed circle for control mice (n = 16); cross for mice transplanted with BaF3 cells (n = 15–16). The graphs are the result of two independent in vivo experiments pooled together. Insets indicate the Pearson correlation coefficient and the corresponding p-value. (TIFF) [file pone.0037971.s003.tif]

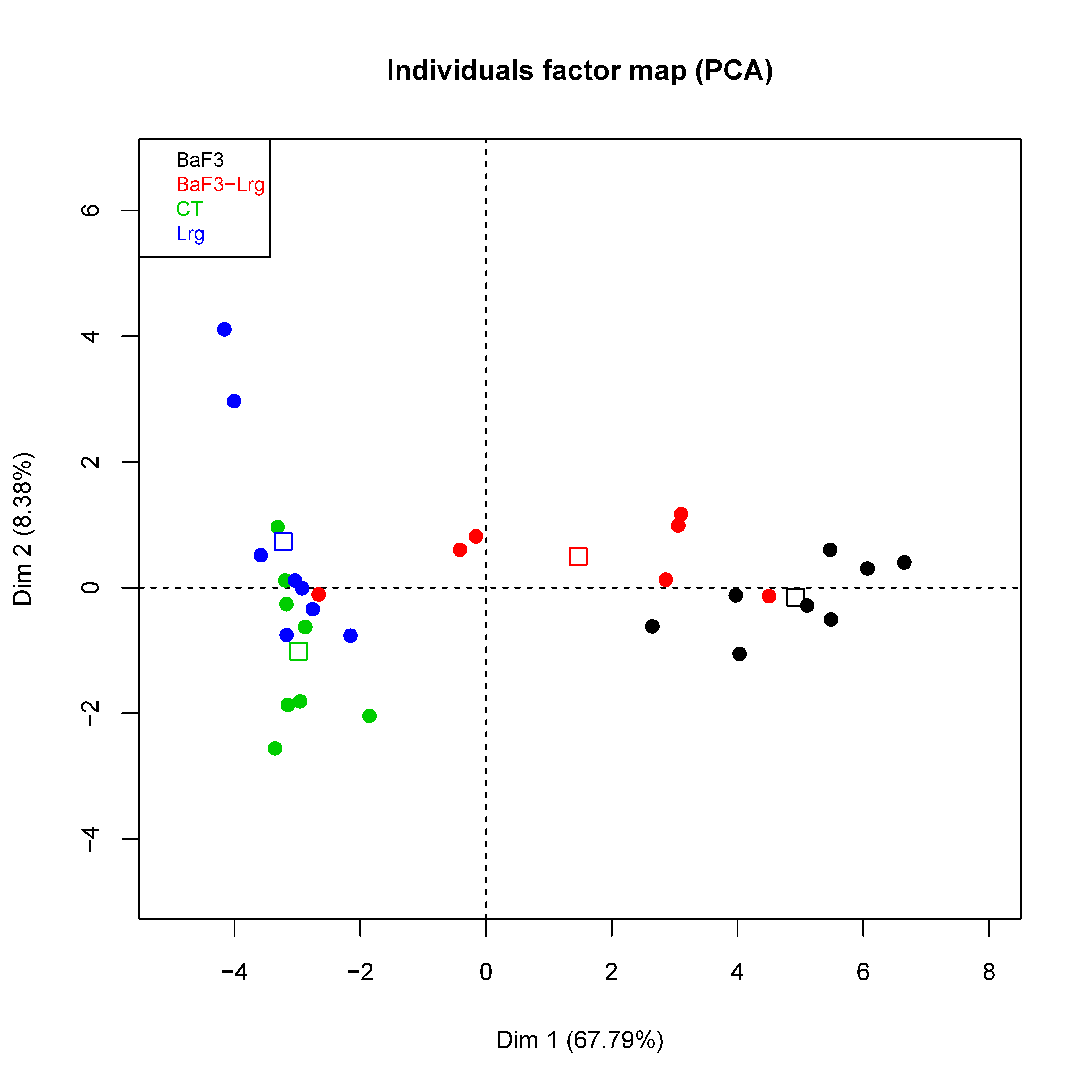

Supplement: Figure S4 — Unsupervised analysis of the supplementation with Lactobacillus reuteri 100-23 and Lactobacillus gasseri 311476. The principal component analysis takes into account lactobacilli levels, muscle weight, muscle atrophy marker expression (tibialis and gastrocnemius), and plasma inflammatory markers. CT = control mice; Lrg = mice receiving lactobacilli; BaF3 = mice transplanted with BaF3 cells; BaF3-Lrg = mice transplanted with BaF3 cells and receiving lactobacilli. (TIFF) [file pone.0037971.s004.tif]

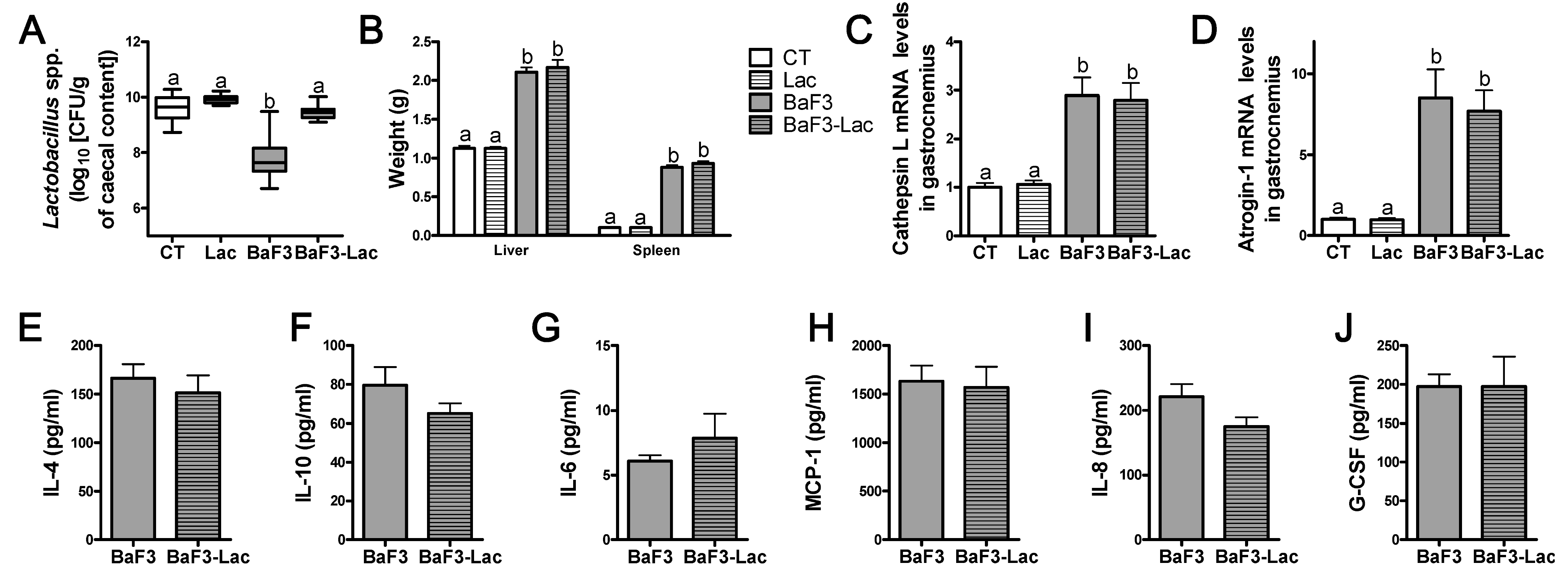

Supplement: Figure S5 — Lactobacillus acidophilus NCFM supplementation does not blunt cachexia. A. Lactobacillus spp. levels in control mice (CT), in mice receiving L. acidophilus NCFM (Lac), in mice transplanted with BaF3 cells (BaF3) and in mice transplanted with BaF3 cells mice and receiving L. acidophilus NCFM (BaF3-Lac). B. Liver and spleen weight. C–D. Atrophy marker expression (Atrogin-1 and Cathepsin L) measured in the gastrocnemius muscle. E–J. Plasma levels of interleukin 4 (IL-4), interleukin 10 (IL-10), interleukin 6 (IL-6), monocyte chemoattractant protein 1 (MCP-1), interleukin 8 (IL-8) and granulocyte colony-stimulating factor (G-CSF). N = 8–10. Data with different superscript letters are significantly different (p<0.05). (TIFF) [file pone.0037971.s005.tif]
